# Commentary: The myth of cognitive agency: subpersonal thinking as a cyclically recurring loss of mental autonomy

**DOI:** 10.3389/fpsyg.2018.01401

**Published:** 2018-08-20

**Authors:** Jonna Vance

**Affiliations:** Department of Philosophy, Northern Arizona University, Flagstaff, AZ, United States

**Keywords:** self-model, narrative, mindwandering, dreaming, sensorimotor control

Metzinger's self-model theory (SMT) provides a rich and flexible framework for theorizing about the self. This commentary discusses how narrative conceptions of the self can be developed within SMT. Against many narrative conceptions of the self, I argue that a substantial proportion of narrative self-representations occur without words, during nocturnal rapid eye movement (REM) dreaming. I also discuss how to situate self-narratives in the overall architecture of SMT.

SMT has two core components. A *phenomenal self-model* (PSM) is a representational structure, the contents of which are the consciously experienced self. A *phenomenal model of the intentionality relation* (PMIR) is a model of an organism's cognitive system as it is currently directed at an intentional object. On SMT, an organism must have a PSM and PMIR in order to have a conscious first person-perspective. According to SMT, our sense of self arises from representational structures modeling aspects of our own cognitive systems (Cf. Metzinger, 2003). PSM and PMIR are two especially important layers (or sets of layers) of self-representation. However, an organism's overall self-model is composed of many layers, with different types of self-related content at the various layers.

In a different strand of research on the self, a number of theorists claim that narratives hold a place of central importance to our sense of self (Dennett, 1991; Gazzaniga, 1995; Schechtman, 2007; Damasio, 2012; Adler et al., 2017). On a narrative conception of the self, self-narratives organize our self-understanding and help us navigate through life. “Narrative” in this literature is usually not rigorously defined. Instead, paradmigmatic examples are used to illustrate the concept. Examples focus especially on stories and novels (Dennett, 1991; Schechtman, 2007). This focus suggests that, necessarily, narratives are composed of words. However, as we'll see below, “narrative” can be used more broadly.

Metzinger typically does not emphasize the concept of narrative in self-representation. His development of SMT has focused especially PSM and PMIR in the senses described above, which make no essential reference to narrative. However, Metzinger (2013) relates SMT to the concept of an automatic narrative (p. 5). Automatic narrative occurs while the brain's default network is active (for more on the default network, see Raichle et al., 2001). For example, REM dreaming exhibits automatic self-narratives.^1^ Metzinger conceptualizes dreaming in terms of loss of several kinds of mental autonomy: *attentional agency* (the ability to control one's focus of attention) and *cognitive agency* (the ability to control goal/task-related deliberative thought (p. 2). Dreaming also involves loss of *veto autonomy*: the personal-level ability to voluntarily suspend or inhibit an action (p. 4). Metzinger (2013) argues that the transition from subpersonal cognition (e.g., dreaming) to personal-level cognition (e.g., deliberate storytelling) is enabled by the activation of a key part of the PSM: an epistemic agent model (EAM), which is a “global model of the cognitive system as an epistemic agent” (p. 1). He provides empirical evidence that mental autonomy is the exception in mental life.

Why are dreams *narratives*? Metzinger provides little on this score. Here I offer several reasons to think that many REM dreams are self-narratives. There is evidence that REM dreams exhibit canonical story elements and structure. Using Mandler and Johnson's (1977) account of story grammar, Cipolli and Poli (1992) found that REM-dream reports given during nocturnal awakenings by patients in a sleep laboratory contained canonical story elements (characters, settings, themes) and story event-structures (initial action, reaction, resolution toward a goal). In addition, mind-wandering and dream narratives involve activation of many brain areas used during mentally autonomous storytelling (Pace-Schott, 2013). Hobson et al. (2000, p. 799) claim that, in REM sleep, “Dreams create story lines to explain and integrate all the dream elements in a single confabulatory narrative,” and they characterize this is one of nine “remarkably consistent…features” across numerous empirical studies.^2^ By contrast, non-REM dreams are often chaotic and lack many of the hallmarks of narrative form (Hobson, 1988; Hobson et al., 2000).

The claim that REM dreams typically involve narratives is controversial (Hobson, 1988). However, at least two significant points of controversy can be avoided here. Regarding the first controversy, one could argue that narratives necessarily are composed of words (Fivush and Merrill, 2016). Many REM dreams involve sequences of images without words. On this objection such dreams could not be narratives. However, this objection is flawed: narratives do not require words. For example, wordless sequences of images in dramatic films constitute narratives (Cumming et al., 2017). Film sequences contain canonical story-elements: characters, settings, actions, themes. And they have story-structure: temporal, causal progression of actions and reactions, sometimes with beginning-middle-end. Second, note that claiming that dreams involve *automatic* narrative is neutral on at least one important point of controversy. Dennett (1991) argues that dreams do not typically involve deliberate and intentional processes of personal-level authorial intent (see also Windt, 2014, 2015). Since automatic narratives also do not involve deliberate and intentional processes of personal-level authorial intent, claiming that dreams involve automatic self-narratives is consistent with Dennett's point.

Why are dreams *self*-narratives? Many dream experiences proceed from the first-person perspective (Llinás and Paré, 1991; Fox et al., 2013). In SMT, experience from the first-person perspective is a form of self-representation. So, when dreams are narratival from the first-person perspective, they are a form of self-narrative. Self-representation in dreams can then be further enriched by drawing on autobiographical memories (Cavallero and Cicogna, 1993; Hobson et al., 2000) and through the experience of the self as a rational agent, as in lucid dreams (Windt and Metzinger, 2007) through the activation of an EAM (Metzinger, 2013). Recall that on SMT, an organism's overall self-model is arranged in a complex network of layers, where the PSM and PMIR are two such layers (or sets of layers). It is worth asking how we should situate the notion of automatic self-narrative within the architecture of SMT. One plausible option is that some layers of one's overall self-model are narrative self-representation layers and others are non-narrative self-representation layers. For example, it could be the case that one's synchronic PSM representations (which represent the self at a time) exist at a non-narrative layer (or layers) while one's diachronic self-representations (which represent the self over time) exist at other layers, at least some of which are self-narrative layers. However, there are other options. For example, layers within an overall self-model might not be neatly separable as narrative and non-narrative layers. In general, it is difficult to delineate the types and order of layers in brain networks (Vance, 2015). More work is needed to clarify the place of self-narratives in the overall architecture of SMT. (For more discussion of the layers in the overall self-model, though not in terms of self-narrative, see Metzinger, 2003; Metzinger and Gallese, 2003).

The approach to self-narrative in SMT recommended here can be usefully applied to a widely-used distinction between the “minimal self” and “narrative self.” In a representative characterization of the distinction, Gallagher describes the minimal self as “a consciousness of oneself as an immediate subject of experience, unextended in time” and the narrative self as “a more or less coherent self (or self-image) that is constituted with a past and a future in the various stories that we and others tell about ourselves” (Gallagher, 2000). From the perspective of SMT, this way of drawing the distinction is a mistake. The synchronic content of a PSM is a minimal self(-model). But Gallagher's characterization of the narrative self focuses too much on conscious, personal-level storytelling as a distinctive feature of the narrative self. As we saw above, automatic self-narratives are a far more typical mode of self-narrative, and they are often sub-personal, non-linguiform representations. Relatedly, Metzinger (2013)'s arguments raise a challenge for narrative conceptions of the self on which personal-level self-narratives are the key to self-understanding (e.g., Dennett, 1991; Schechtman, 2007). Personal-level self-narratives require activation of EAM, according to Metzinger. Using data from sleep science, Metzinger (2013, p. 6) conservatively calculates that more than 80% of our REM sleep consciousness lacks mental autonomy (see also Nielsen, 2000; Hobson, 2003). On the broad approach to self-narrative I am recommending, much of our thought in REM dreams is automatic self-narrative. Thus, narrative conceptions of the self should be applied to automatic self-narratives that occur during REM dreaming. Indeed, narrative conceptions of the self should emphasize automatic self-narratives as central components of self-narrative mental processing.

With an expanded conception of self-narrative, one can find self-narrative representations in other mental processes, beyond deliberate storytelling and dreaming. For example, sensorimotor control arguably exhibits self-narrative in an expanded sense. Motor-control systems represent one's active body across time, with beginning-middle-end structure, and within a specific setting (including environmental dynamics). In addition, on standard approaches in sensorimotor psychology, motor commands serve as initiating events, internal and external noise and environmental dynamics provide conflict, and the sensorimotor system must respond to find resolution and achieve its task goal (Todorov and Jordan, 2002; Vance, 2017). As such, motor control representations exhibit key aspects of story-structure, where the main character in the story is oneself.

Relatedly, on the expanded conception, self-narrative plays important causal roles in organisms' overall functioning. This is important to note, since the causal role of self-narrative has been denied (Bickle, 2003). Conceiving of REM dreaming and mind-wandering as both being forms of automatic self-narrative realized by the brain's default network, Metzinger notes that dreaming and “mind-wandering would then be a sort of baseline activity serving to maintain a minimal level of arousal and functional continuity, the default mode of narrative self-modeling, a permanent mechanism of re-encoding and synaptic stabilization, constructing a *domain-general* functional platform enabling long-term motivation and future planning” (p. 6). In playing this stabilizing role, automatic self-narratives make a crucial contribution to the organism's behavior. By broadening the notion of self-narrative to the self-modeling arc of motor control, one sees a further causal role for self-narrative. Overall, the broad approach to self-narrative within SMT recommended here connects self-narratives with many self-modeling systems not included in standard linguistic characterizations of self-narrative. As such, the approach provides an important suggestion for narrative-self researchers to consider and to develop further.

## Author contributions

The author confirms being the sole contributor of this work and approved it for publication.

### Conflict of interest statement

The author declares that the research was conducted in the absence of any commercial or financial relationships that could be construed as a potential conflict of interest.

## Footnotes

^1^In addition to nocturnal REM dreams, many daydreams (mindwanderings) arguably exhibit self-narrative. Due to limitations of space, I omit discussion of mindwandering here.

^2^On this point, Hobson et al. (2000) cite Foulkes (1985); Hobson (1988); Hunt (1991); Blagrove (1992); Cipolli and Poli (1992); Cipolli et al. (1998); and Montangero (1991).
